# Stress related epigenetic changes may explain opportunistic success in biological invasions in Antipode mussels

**DOI:** 10.1038/s41598-018-29181-4

**Published:** 2018-07-17

**Authors:** Alba Ardura, Laura Clusa, Anastasija Zaiko, Eva Garcia-Vazquez, Laura Miralles

**Affiliations:** 1 Department of Functional Biology, University of Oviedo, C/Julian Claveria s/n, 33006 Oviedo, Spain; 2 Coastal and Freshwater Group, Cawthron Institute, Private Bag 2, Nelson, 7042 New Zealand; 3 Marine Research Institute, Klaipeda University, H. Manto 84, Klaipeda, 92294 Lithuania

## Abstract

Different environmental factors could induce epigenetic changes, which are likely involved in the biological invasion process. Some of these factors are driven by humans as, for example, the pollution and deliberate or accidental introductions and others are due to natural conditions such as salinity. In this study, we have analysed the relationship between different stress factors: time in the new location, pollution and salinity with the methylation changes that could be involved in the invasive species tolerance to new environments. For this purpose, we have analysed two different mussels’ species, reciprocally introduced in antipode areas: the Mediterranean blue mussel *Mytilus galloprovincialis* and the New Zealand pygmy mussel *Xenostrobus securis*, widely recognized invaders outside their native distribution ranges. The demetylathion was higher in more stressed population, supporting the idea of epigenetic is involved in plasticity process. These results can open a new management protocols, using the epigenetic signals as potential pollution monitoring tool. We could use these epigenetic marks to recognise the invasive status in a population and determine potential biopollutants.

## Introduction

Epigenetics, or chemical signatures of DNA that can activate or silence genes (methylation^1,2^ or histones (acethylation/de-acethylation^3,4^)), are rapid mechanisms of response of Eukaryotes to environmental challenges^5,6^. Perhaps the greatest challenge that a marine organism may undertake is to be transferred to a distant location mediated by a rapid vector, for example associated to human transport, much faster than the natural dispersal rate. During the travel, an organism may survive under adverse conditions^7–10^, and in the location of introduction it may need to adapt to a new environment that likely differs from the native one.

In some cases, translocated species are able to adapt easily to the recipient ecosystem, proliferate and may even become invasive^11–13^. The role of epigenetics in biological invasion processes, in particular methylation, has been discussed earlier^14^, but was deeply studied only in a few cases. Higher epigenetic than genetic variation reported in introduced populations of plants^15^ and vertebrates^16^ suggests that epigenetics may compensate for reduced genetic diversity in incursion areas, e.g. due to founder effects^17^. Decreased global methylation has been proposed as a mechanism for enhancing plasticity, thus tolerance to new conditions, in recent invertebrate invasion events^18^. Nevertheless, this phenomenon needs to be further explored, as it has enormous ecological implications. It could explain unpredictable invasive success of some species able to acquiring novel traits and tolerances in the recipient ecosystem. Niche shifts that facilitate expansion of invaders^19,20^ could be at least partially explained through epigenetic mechanisms that are more rapid than genetic adaptation by natural selection. However, as long as the introduced population settles down in the recipient ecosystem and leaves the expansive phase, extra plasticity would no longer be necessary and the levels of methylation are expected to reverse to species’ normal state, approaching those and be similar to those of native populations. This phenomenon would be reflected in increased methylation in older introduced populations versus more recent ones. In other words, generalized hypomethylation would be a signature of early (versus old) introductions^18^.

The same rationale could expectedly be applied to expansive populations colonizing new niches within the native distribution range, or facing environmental disturbance. Methylation is a fast response to environmental stress and can be inheritable^21–24^. Pollution^25,26^ and salinity^27^ are examples of stressors in marine environments that can induce DNA hypomethylation. The early phase of biological invasions could be also viewed as a type of stress, as it corresponds to settling and expanding into a new environment.

Invasive species are thought to be tolerant to a wide range of stressors, including pollution, and may occupy niches that native species are not able to exploit^28,29^. The newly introduced specimens are likely to be exposed to pollution (as settlement often takes place in disturbed areas such as ports) or the recipient area may not be within the optimal environmental range of the species. Therefore, those having better adaptation mechanisms have higher chances of survival, establishing a thriving population and ultimately conquering the ecosystem^28–30^.

In this study, we have tested the hypothesis of decreased global methylation as a mechanism for easing tolerance to stress conditions, including early invasion phases, in two model species: the Mediterranean blue mussel *Mytilus galloprovincialis* and the New Zealand pygmy mussel *Xenostrobus securis*. These species are widely recognized invaders outside their native distribution ranges^31–35^. The two species are quite similar in their biological traits and life style (see below, in material and methods section) and are close phylogenetically, belonging to the same Mytilidae family. Such ecological resemblance allowed us to expect similarity in their adaptation mechanisms and epigenetic responses to environmental changes.

Both species are reciprocally native and introduced populations in antipode locations - (Southern Europe and New Zealand). Two incursions of *M*. *galloprovincialis* seemingly took place in New Zealand: one in the Pleistocene, another - comparatively recently, and likely due to human activities such as maritime traffic^36–39^. The older lineage exhibits approximately 1.4% divergence from the closer north Atlantic clade at 16 S rDNA, while the more recent lineage is 0.3% divergent from north Atlantic populations^37^. Only this second lineage will be taken into account in this study, since the old one introduced in the Pleistocene should be totally naturalized today, thereby not fitting the current objectives.

On the other hand, the first introduction of New Zealand-native *X*. *securis* to Europe is comparatively recent, about three decades ago^40^. This species is currently undergoing the expansion or outbreak phases in incursion areas^35,41^. The rationale of epigenetic response (demethylation in initial stages of invasion) implies that its populations would be less methylated than native or naturalized ones.

## Material and Methods

### The species studied

*Mytilus galloprovincialis* and *Xenostrobus securis* are sessile byssate bivalves, broadcast spawners, highly tolerant to wide salinity and temperature ranges^42^. The species are able to adapt well to highly degraded and polluted habitats such as ports^43^, bioaccumulate different pollutants (for *Mytilus galloprovincialis*;^44,45^ for *Xenostrobus securis*^33,46,47^). The suggested main differences between species are smaller size of *X*. *securis* and its preference for more brackish environments comparing to *M*. *galloprovincialis*^35,48^.

### Sampling sites, specimen collection and stress factors

Mussel specimens were sampled simultaneously from two sites of different environments in south Europe and two in New Zealand, where *M*. *galloprovincialis* and *X*. *securis* are respectively native to and introduced. The summarized information on the sampled populations is provided in Table 1. Two sites from Oceania, New Zealand (Nelson and Havelock, South Island) and two from south Europe (Aviles, Spain and nearby Vidourle, south France) were sampled for Mediterranean blue and pygmy mussels. Adult individuals of the two species were identified *de visu* and taken at random from the sampling sites (at least 15 per population and species). Specimens were preserved in absolute ethanol for further analysis.

**Table 1:** Environmental conditions in the four sampling sites.

Continent	Country	Region	Location	Coordinates	Sampling season	Average annual temperature (°C, min-max)	Annual rainfall (mm)	Ports	Average annual salinity (ppm, min-max)	Environment/human population	Anthropogenic pressure
Europe	Spain	Northwest Spain	Aviles	43°25′16″N, 4°45′11″W	Winter	13.5 (9.9–17.1)	1062	International cargo & fishing port	29.7 (22.8–34.1)	Urban, city of 80,880 inhabitants	Port, substantial industrial pollution, heavy metals
Europe	France	Mediterranean Sea	Vidourle	43°34′42.14′′N, 4°02′34.98′′E	Winter	15.1 (10.4–19.9)	629.1	Small fishing ports and marina	23.9 (20.4–27.3)^88^	Villages of 8,505 Grande-Motte and 3,707 inhabitants Carnon	Eutrophication - Protected area Natura 2000^51^
Oceania	New Zealand	South Pacific Ocean	Havelock (Pelorus Sound)	41.2846° S, 173.7672°E	Winter	12–18	1250	Small fishing port and marina	~30, may be reduced for long periods^89^	Township of 480 inhabitants	Eutrophication, sediment loads from upstream^49,50^
Oceania	New Zealand	South Pacific Ocean	Nelson (South Island)	41°16′15″S 173°17′2″E	Winter	15.1 (11.9–19.8)	994.5	International cargo & fishing port	32–35^25,90^	Urban, city of 65,700 inhabitants	Port, local industries, forestries and farmlands

The sampling locations were chosen to account for difference in salinity preference of the considered species and pollution as environmental stress. Thus, Vidourle (France) is a protected coastal lagoon (included in Natura 2000 network) with brackish water, affected by seasonal inflows from Vidourle river and little anthropogenic influence. The levels of anthropogenic pressures in Vidourle can be compared to Havelock (Pelorus Sound, New Zealand), located in sounds at the mouth of two rivers far from industrial pollution and big urban centres. Both locations, as estuarine habitats, are subjected to eutrophication pressure and freshwater inputs (Havelock^49,50^; Vidourle lagoon^51^). On the other hand, Aviles (Spain) and Nelson (New Zealand) are both international commercial ports with busy shipping traffic, adjacent to regional urban centres and dominated by marine waters (with some flushing effect from local rivers). All sampling locations have low level of exposure to the open sea and resembling tidal ranges, therefore the effect of these natural stress factor was considered negligible and not accounted for in this study. General environmental information (temperature and salinity) for the sampling areas was collected from the online resources (e.g. National Agency of Meteorology, Global Sea Temperature website, ClimaTemps website, National Ports –Spanish ports at http://www.puertos.es/es-es), published national and regional reports and research papers.

Three different stress factors (invasion-related, natural and anthropogenically driven) have been considered. (i) the population age as a proxy for early invasion stress; (ii) salinity stress in relation to reported tolerance ranges for each species; and (iii) habitat degradation, represented by anthropogenic pressures assumed for each location. Each factor was scored as follows:

- Early invasion stress (=population age). Scored 1–5 and relates to the invasion-naturalisation status of the population, where 1 corresponds to native population, 2 - to “old” introduction (>30 years) and therefore supposedly fully naturalized population, 3 - to 30–10-year introduction, 4 - to relatively recent introduction (10–5 years), and 5 - to very recent introductions (<5 years) (Table 1). The introduction age was estimated from literature and official first reports of species detection whenever possible (*X*. *securis* in Europe: 2014 in Aviles versus 1992 in Vidourle). For *M*. *galloprovincialis* in New Zealand a score of 2 was considered, since, although non-native, the Atlantic lineage is suspected to be there for a long time, > 30 years at least^37,52^.
- Salinity stress. Scored 1–4, since the range of differences among sites for this factor was comparatively shorter than for the introduction age.
  For *Xenostrobus securis*, although tolerates salinities from 2 up to 30 or higher^42^, it prefers brackish waters (5 to 28 ppt^53^) and the upper tolerance range of developing larvae is around 18 ppt. Therefore, salinity >30 ppt could be considered as suboptimal (i.e. stressful). For scoring this data, 1 correspond to places with salinity between 5 and 28 ppt., 2 – to places with salinity between 2 and 30 ppt., 3 – to places with salinity sometimes out of range, and 5 – to places with salinity out of range always.
  For *M*. *galloprovincialis*, also tolerant for a wide salinity range, significant salinity stress as measured from heart rate occurs below 22 ppt^31^. Thus <22 ppt was considered salinity stress for this species. For scoring this data, 1 correspond to places with salinity below to 22 ppt., 2 – to places with salinity below to 22 ppt. sometimes during the year, 3 – to places with salinity below to 22 ppt. most of the time, and 5 – to places with salinity below to 22 ppt. always.
- Habitat degradation (=anthropogenic pressure). Scored between 1–4 based on pollution and other factors reported from the studied sampling sites (Table 1), where 1 correspond to protected area, 2 – to areas with continuous sediment loads around, 3 – to port areas with local industries such as forestries and farmlands, and 4 – to port areas with industrial big pollutions like heavy metals.

Mapping vulnerability is most commonly performed by combining multiple indicators into single indices of vulnerability for a given stressor under a given dimension, and then combining multiple indices in order to build an overall, relative estimate of vulnerability^54,55^. These “combinations” are usually simple arithmetic or geometric means, based on the Multiple Attribute Utility Theory (MAUT) that is widely used in economics, engineering, decision science, development studies and, to a lesser extent, social sciences^56–59^. In order to analyze the synergetic effect of considered stress factors on methylation patterns, additive and multiplicative aggregation of stressor scores was applied, following vulnerability assessment framework described by Hinkel (2011). This approach is widely used in the Indicator Based Vulnerability Assessment (IBVA), where aggregation approaches (e.g., weighted additive or multiplicative aggregation) based on MAUT are applied^60^.

### Ethical statement

This study has been carried out on invertebrate species. Since they are reported as invasive in various regions, the materials and clothes employed in sampling and manipulation of individuals were carefully cleaned and disinfected to avoid further dispersion of these organisms or their propagules in the environment. This study adheres to the European Code of Conduct for Responsible Research.

### DNA analysis

Total DNA was extracted from the foot muscle of collected specimens using the E.Z.N.A Mollusc DNA kit (IOMEGA, Bio-Tek) following manufacturer’s instructions. DNA samples were stored at 4 °C until analysis conducted. Aliquots were long-time stored at −20 °C. DNA quantification was carried out by fluorometric methodology with Qubit® 2.0, and normalized to 100 ng/µl.

MSAP (methylation-sensitive amplified polymorphism) methodology was applied to measure polymorphism in DNA methylation patterns. The global methylation was analysed following Díaz-Freije *et al*.^61^. This protocol was also described in detail in Ardura *et al*.^18^. Briefly, two aliquots of 100 ng DNA per sample were treated separately with *EcoRI*/*HpaII* and *EcoRI/MspI*. The enzymes (*MspI* and *HpaII*) recognize and cleave CCGG target sequences, depending on the methylated status in the inner and/or outer C^62^.

The resulting DNA fragments were ligated with linkers and PCR amplified using two combinations of primers: [*EcoRI*-AAG, *HpaII*-TCC] and [*EcoRI*-AAG, *HpaII*-TAC]. *HpaII* primers were end-labeled using 6-FAM reporter molecule^63^. PCR products were loaded with GeneScan GS-500 LIZ3130 size standard into an ABI Prism 3100 Genetic Analyzer (Applied Biosystem).

Fragment analysis and AFLP scoring were performed with GeneMapper v.4.0 software (Applied Biosystem). The following settings were applied for quality AFLP reading: analysis range, 50–500 base pairs (bp); minimum peak height, 50 relative fluorescence units; pass range for sizing quality: 0.75–1.0; maximum peak width: 1.5 bp; maximum peak height ratio: 1.8 (higher peaks were removed); normalization method: sum of signals. To confirm AFLP reproducibility five *X*. *securis* samples were analysed again following the same protocols.

### Data analysis

Individual and population MSAP profiles were analysed with the R package msap v.3.2.2^64^. The software yields a score matrix according to the methylation state, using the four patterns from presence-absence matrices obtained with the *EcoRI-HpaII* and *EcoRI-MspI* primer combinations (Type I to IV) described by Salmon *et al*.^62^.

Type IV variation could be due to high methylation status but also to mutations in restriction sites, thus real methylation state cannot be specified^62^. Following Fulnecek and Kovarik^65^, we have considered type II and III together as methylated loci. Finally, type I restriction sites are not methylated. The global methylation level was measured as the proportion of methylated loci (Types II and III) over the scorable loci (Types I, II and III), as in Nicotra *et al*.^66^ and Ardura *et al*.^18^.

In methylation-susceptible loci (MSL) the observed proportion of methylated states across all samples exceeded a user-defined error rate-based threshold (ERT; 5% by default). The rest of loci were categorized as non-methylated (NML). Only polymorphic fragments with at least two occurrences of each state were used for subsequent analysis (Herrera & Bazaga 2010). MSL were used to assess epigenetic variation, and NML – to assess genetic variation since their banding pattern depends exclusively on changes in the sequence at the restriction target sites.

The R package msap v.3.2.2^64^ and GenAlEx software^67,68^ were employed for the following analyses. For both MSL and NML the amount of overall variation was estimated using the Shannon diversity index (I). Differences between Shannon’s indices between MSL and NML were tested with the Wilcoxon rank sum test with continuity correction (W). The epigenetic (MSL) and genetic (NML) differentiation among populations and between pairs of populations were assessed by means of ɸ_ST_ values, and principal coordinates analyses (PCoA) followed by analysis of molecular variance (AMOVA)^69^.

Software PAST^70^ was employed to compare the mean proportion of methylated loci per individual between populations using conventional t-tests, after testing variance equality through F test. Analysis of residuals was performed and normality was assessed using Shapiro-Wilk test. A Principal Components Analysis (PCA) was carried out with the same software to identify the relative contribution of the different stressors to the dataset variance, after checking compliance of the necessary conditions in the dataset: applying correlation function and 0.7 eigenvalue cutoff. Correlation between methylation and stressors was calculated using Pearson’s r after checking normality, with 0.05 significance threshold and applying Bonferroni correction whenever relevant.

## Results

In total 15, 21, 25 and 29 X. *securis* and 24, 26, 10 and 23 *M*. *galloprovincialis* individuals were analysed from Havelock, Nelson, Vidourle and Aviles populations respectively. Sequences of 16 S rDNA of *M*. *galloprovincialis* (GenBank accession numbers MF463020-MF463027 for the haplotypes found in these samples) revealed that only 15 individuals from Nelson and 13 from Havelock belonged to the Atlantic lineage (Supplementary Figure 1). MSAP analysis was performed only on Atlantic-origin *M*. *galloprovincialis* and all the *X*. *securis* individuals. *M*. *galloprovincialis* individuals of the old Pacific introduced lineage were not considered.

For *X*. *securis*, 432 AFLP loci (Supplementary Table 1) were found in the populations analysed: 219 MSL (96% polymorphic) and 213 NML (100% polymorphic). For *M*. *galloprovincialis* 278 AFLP loci were found (Supplementary Table 2), 87 MSL (76% polymorphic) and 191 NML (99% polymorphic). The two species exhibited high diversity at MSL and NML in the four studied populations. In *M*. *galloprovincialis*, Shannon’s Diversity Index was 0.525 (SD: 0.158), and 0.257 (SD: 0.150) for MSL and NML respectively. Statistical significant difference was confirmed by Wilcoxon rank sum test with continuity correction, W = 13882 (P < 0.00001). In *X*. *securis*, Shannon’s Diversity Index was 0.575 (SD: 0.135) and 0.285 (SD: 0.159) for MSL and NML respectively (W = 40392.5, P < 0.0001).

In the global analysis, taking each population as a unit, both MSL and NML differed significantly among the four studied populations of the two species, with significant Ф_ST_ values for the two types of loci (Table 2). These values represent genetic differentiation between populations based on different alleles (or methylation patterns) in the loci analysed. The four ANOVAs (for the two species and locus type from methylation sensitiveness) were statistically significant, with p < 0.01 in all cases (Table 2).

**Table 2:** Global statistical analysis of methylation sensitive loci (MSL) and non-methylated loci (NML) in the two mussel species analysed.

**MSL**					**NML**				
**Source**	**d**.**f**.	**SSD**	**MSD**	**Variance**	**Source**	**d**.**f**.	**SSD**	**MSD**	**Variance**
*Mytilus galloprovincialis*									
among groups	3	63.63	21.21	0.7155	among groups	3	68.17	22.72	0.6117
within groups	50	582.2	11.64	11.64	within groups	50	727.2	14.54	14.54
Total	53	645.9	12.19		Total	53	795.4	15.01	
Ф_ST_ = 0.05789 (P = 0.0081)					Ф_ST_ = 0.04036 (P < 0.0001)				
*Xenostrobus securis*									
among groups	3	931	310.3	12.11	among groups	3	319.3	106.4	4.094
within groups	88	3370	38.3	38.3	within groups	88	1276	14.49	14.49
Total	91	4301	47.27		Total	91	1595	17.53	
Ф_ST_ = 0.2403 (P < 0.0001)					Ф_ST_ = 0.2203 (P < 0.0001)				

**Figure 1:**
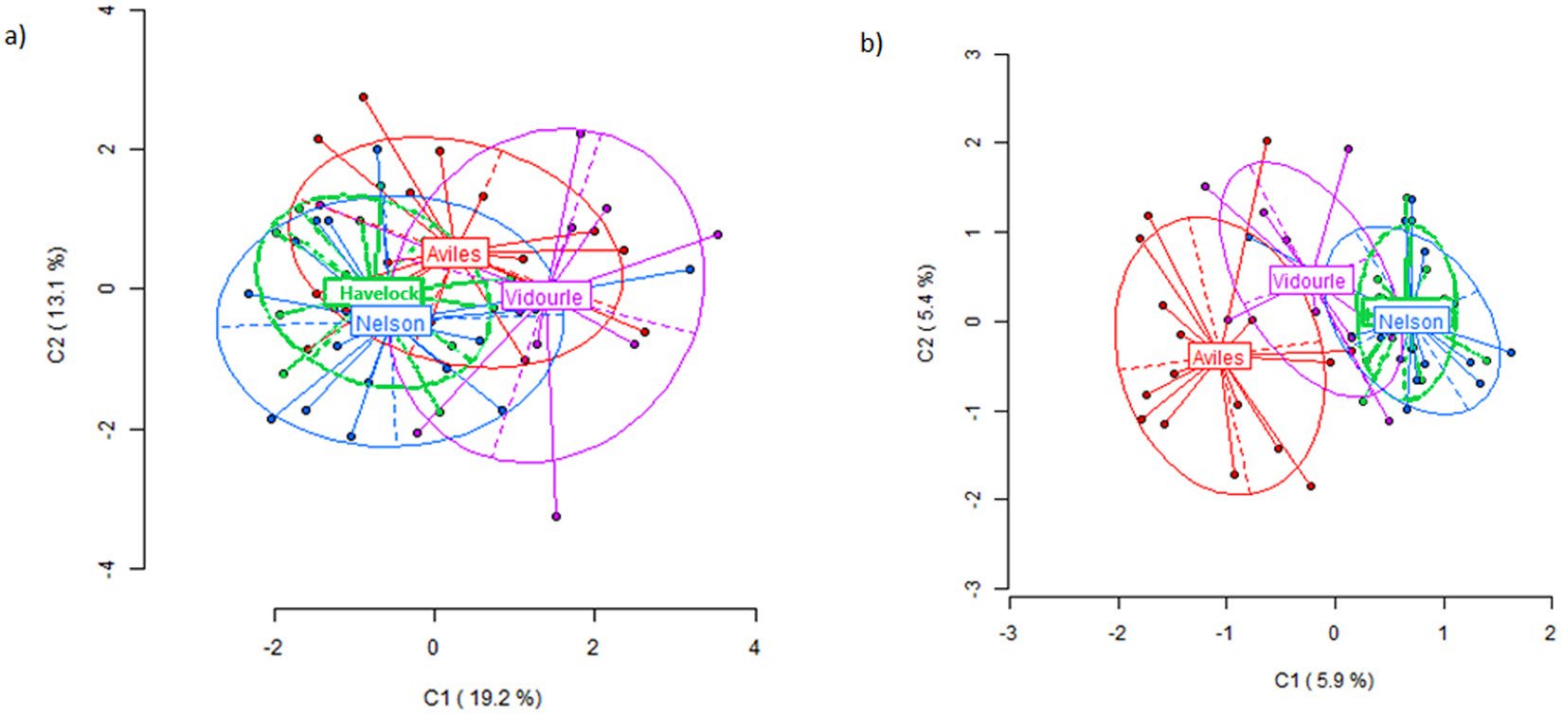
Two-dimensional visualization of the Principal Component Analysis (PCoA) of the detected methylation patterns in *Mytilus galloprovincialis*. The individuals of each population are represented by Aviles, Vidourle, Havelock and Nelson. (**a**) The epigenetic variation (methylation-sensitive loci -MSL). (**b**) The genetic variation (no methylated loci, NML).

**Figure 2:**
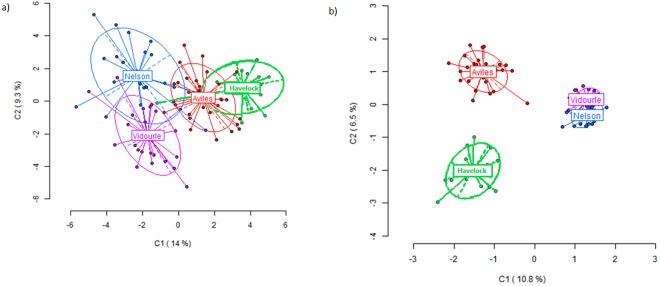
Two-dimensional visualization of the Principal Component Analysis (PCoA) of the detected methylation patterns in *Xenostrobus securis*. The individuals of each population are represented by Aviles, Vidourle, Havelock and Nelson. (**a**) The epigenetic variation (methylation-sensitive loci-MSL). (**b**) The genetic variation (no methylated loci, NML).

In terms of genetic differentiation (variation at NML), native-native and introduced-introduced pairwise Ф_ST_ were both significant for *X*. *securis*, and the absolute value was greater between natives (Ф_ST_ = 0.262 and 0.181 respectively, both with *p* << 0.001). For *M*. *galloprovincialis* only native-native pairwise Ф_ST_ was significant (Ф_ST_ = 0.036, *p* << 0.001), while introduced-introduced comparison was not (Ф_ST_ = 0.013, P = 0.914). For *M*. *galloprovincialis*, differences among populations, although significant between native populations, did not separate completely the four populations for any type of loci. In PCoA analysis, more apparent differentiation of the Mediterranean (Vidourle) population was observed for MSL (Fig. 1a) while for NML the apparently more differentiated population was the Atlantic one (Aviles) (Fig. 1b). For *X*. *securis*, the populations were clearly separated based on MSL (Fig. 2a), while based on NML Aviles and Havelock populations were clearly apart but Nelson and Vidourle were more overlapped (Fig. 2b).

**Figure 3:**
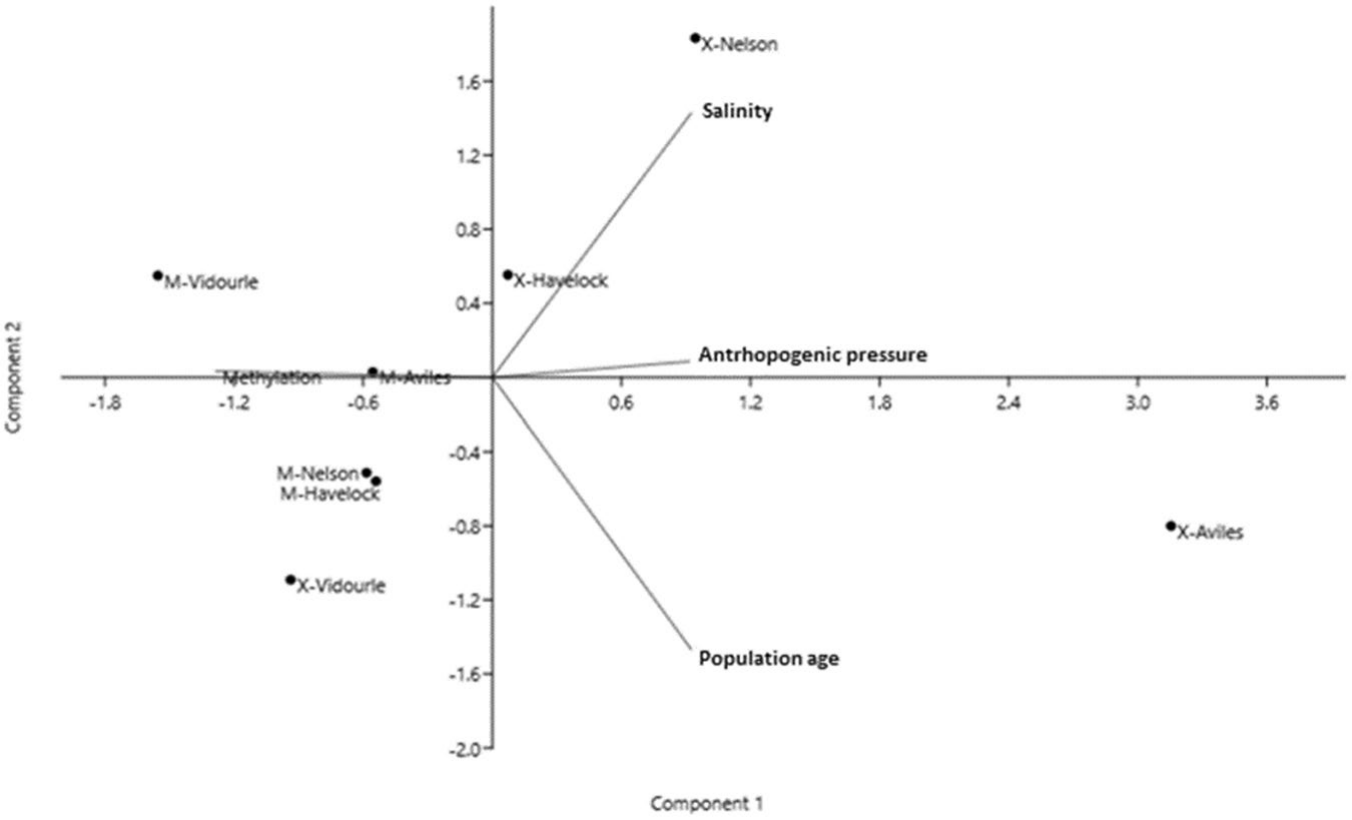
Scatter plot of the stressors and global methylation derived from Principal Components analysis. Mussel populations are indicated as M = *Mytilus galloprovincialis*, X = *Xenostrobus securis*.

Given significant among-population differences in epigenetic variation in the reciprocally antipode mussels analysed here, the proportion of each type of methylation status for MSL loci was also analysed (Table 3). Results revealed a similar mean proportion of each type of locus (type I to IV) in the two species, with a slightly higher proportion of fully methylated and/or mutation in restriction sites (type IV) for *X*. *securis* than for *M*. *galloprovincialis*. The mean proportion of methylated loci in the populations analysed exhibited similar range of variation in the two species, being 0.64–0.7 in M. *galloprovincialis* and 0.57–0.69 in *X*. *secures*. The ranges were quite similar for the global population methylation calculated considering the population as a unit (Table 4). The stressor scores were different in the analysed populations and species (Table 4), depending on the species and population characteristics as explained above. In the PCA, the components 1 and 2 together contributed with >76% variance (Table 5). The relative contribution of stressors to the total variance was higher for population age in the component 1, salinity in the component 2, and anthropogenic pressure in the component 3 (Table 5), methylation being located opposite to the three stressors in the scatter plot of the principal component analysis (Fig. 3).

**Table 3:** Mean proportion (SD in parenthesis) of different methylation types in methylation-sensitive loci for the eight mussel populations analysed in this study.

**Region**	**Populations**	*Mytilus galloprovincialis*				*Xenostrobus securis*			
		**Type I**	**Type II**	**Type III**	**Type IV**	**Type I**	**Type II**	**Type III**	**Type IV**
SE	Aviles	0.097 (0.032)	0.127 (0.027)	0.135 (0.041)	0.641 (0.123)	0.123 (0.029)	0.083 (0.018)	0.105 (0.091)	0.713 (0.027)
SE	Vidourle	0.093 (0.034)	0.118 (0.031)	0.121 (0.035)	0.668 (0.049)	0.098 (0.022)	0.119 (0.033)	0.108 (0.031)	0.675 (0.047)
NZ	Nelson	0.108 (0.039)	0.125 (0.021)	0.137 (0.055)	0.63 (0.061)	0.076 (0.019)	0.048 (0.014)	0.088 (0.018)	0.788 (0.033)
NZ	Havelock	0.094 (0.045)	0.120 (0.047)	0.117 (0.049)	0.669 (0.081)	0.103 (0.023)	0.053 (0.018)	0.113 (0.017)	0.731 (0.026)

SE and NZ are south Europe and New Zealand, respectively.

**Table 4:** Stressor scores: for population age, salinity and anthropogenic pressure; global methylation (at population level) and mean individual methylation (SD in parentheses), in the eight analysed mussel populations.

Species	Population	Stressors			Total stressor score		Methylation status	
		Population age	Salinity	Anthropogenic pressure	Summative model	Multiplicative model	Global methylation	Mean individual methylation (SD)
*Mytilus galloprovincialis*	Aviles	1	1	4	6	4	0.694	0.693 (0.091)
	Havelock	2	1	2	5	4	0.659	0.674 (0.098)
	Nelson	2	1	3	6	6	0.692	0.646 (0.088)
	Vidourle	1	2	1	4	2	0.716	0.697 (0.117)
*Xenostrobus securis*	Aviles	5	3	4	12	60	0.565	0.573 (0.113)
	Havelock	1	2	2	5	4	0.617	0.618 (0.075)
	Nelson	1	4	3	8	12	0.641	0.633 (0.058)
	Vidourle	3	1	1	5	3	0.686	0.691 (0.083)

**Table 5:** Principal Component (PC) analysis of the stressors considered and global methylation in the eight mussel populations examined, showing the Eigenvalue (0.7 cutoff), % variance explained by each PC, and load of each variable in each PC.

	PC 1	PC 2	PC 3	PC 4
Eigenvalue	2.154	0.911	0.722	0.213
% variance	53.84	22.77	18.05	5.34
Population age	−0.627	0.016	0.211	0.749
Salinity	0.451	−0.716	−0.259	0.465
Anthropogenic pressure	0.451	0.696	−0.324	0.454
Methylation	0.447	0.042	0.885	0.124

In the eight populations, no single stressor had significant correlation with global or mean individual methylation (data not shown). The sum of stressor scores for each population (Table 4) was negatively correlated with the mean individual methylation (r = −0.804, 6 d.f., P = 0.016) and the global methylation (r = −0.786, 6 d.f., P = 0.021), both significant after Bonferroni correction. For multiplicative model (Table 4), the correlation was also significant for both mean (r = −0.791, P = 0.019) and global methylation (r = −0.800, P = 0.017). Pairwise effects of stress factors were also assessed for summative and multiplicative models, applying Bonferroni for statistical significance. For multiplicative model, the results have shown significant correlation of salinity and anthropogenic pressure combination with mean methylation; salinity and population age combination with global methylation, and population age and anthropogenic pressure with either global and mean methylation (data not shown). While for summative model, only the combinatory effects of anthropogenic pressure and population age with global methylation were significant (r = −0.783, P = 0.022).

## Discussion

The results presented here reveal, for the first time, the evidence of epigenetic variation, through changes in DNA methylation, between two reciprocally introduced marine related species with similar biological traits, life style and invasive capacity. Our findings suggest that this variation can be explained by invasion-related stress factors including population age. Epigenetics being involved in the process of establishment in biological invasions had been demonstrated already^6,18,71^. Previous study from the same authors (Ardura *et al*.^18^) hinted that the expected influence of environmental stressors on methylation could be detected in native populations without the “invasive” signature. For this reason, the idea of epigenome analysis of donor and introduced populations together with the population status assessment (early invasion phases) and environmental stress factors was challenged here. The results of this new study supported that hypothesis, suggesting early invasion stage as a stressor, to be added to other environmental stressors as a trigger to epigenetic changes.

Expansion into a new territory would require mobilization of physiological resources, and demethylation may facilitate the process by unblocking genes previously silenced in native (optimal) conditions. In molluscs, although previous studies have reported that highly expressed genes show the highest degree of methylation^72–74^ reported a negative relationship between DNA methylation and expression of certain homeobox (hox) genes during embryonic development in *C*. *gigas*. This implies that DNA hypomethylation is potentially linked to the transcription of genes potentially involved in phenotypic plasticity and adaptation^75^. Thus, demethylation in response to exposure to pollutants was reported for example in humans^22^ and fish^26^. Karan *et al*.^27^ described hypomethylation associated to salinity stress in rice, and Lu *et al*.^76^ reported changes in methylation patterns induced by salt treatment in cotton. In our study, single stressors did not correlate significantly with decreased methylation pattern at population level, but rather had a combinatory effect. The stress factors would be acting here mainly in a multiplicative way, thus suggesting a certain synergetic effect. In fact, the effect of co-exposure to different stressors is rarely summative, since their combined actions may synergistically multiply the negative effects on the organism or, conversely, one stressor may mask the effect of other stressor (see for example Manti & D’Arco^77^ for a review). The same may apply to epigenetic responses to multiple stressors, as experimentally proven on different model and non-model species (reviewed inanalysed Vandegehuchte & Colin^78^). However, the present study is observational and cause-effect associations cannot be proven from it. Experiments subjecting the two species to different levels and combinations of the stressors could be suggested for further empirical verification.

The levels of methylation and epigenetic variation, as well as their distribution in the genome are very diverse within and among phylogenetic groups, despite to be an ancient evolution mechanism^75^. It has been reported earlier, that epigenetic mechanisms may facilitate the establishment and spread of invasive species and important pests^79^ (reviewed in Hawes *et al*.^80^), and we could expect diverse levels of methylation signalling this. The global DNA methylation level reported in this study for the two analysed species (>50%, up to 70% in some cases) was considerably higher than the level reported earlier in other molluscs. For example, Zhikong scallop *Chlamis farreri* exhibited <30% global methylation in different tissues including muscle with dominant Type II (internal cytosine methylated) loci^81^. However, our results are not exceptional. Based on the current results, in both *M*. *galloprovincialis* and *X*. *securis* Type III methylation (hemimethylated loci) was more prevalent than Type II, and this is consistent with findings from recent epigenetic study of *Octopus vulgaris*^82^. Using the same method, high and variable methylation levels were estimated in other organisms: 70% in *Hordeum vulgare*^83^, 15.49–46.10% in *Arabidopsis thaliana*^83^, ∼60% in *Lagopus lagopus scotica*^83^, 23.3–33.4% in *Salmo trutta*^83^ and 63.5–73% in *Ficopomatus enigmaticus*^18^. This suggests that different organisms in general and mollusc species, in particular, likely exhibit different patterns of global methylation even at intraspecific level.

Huang *et al*.^83^ obtained evidence that suggest that stress-induced DNA methylation variation can contribute to the rapid acclimatization to sudden environmental changes at the individual level, and the increased intrapopulation variation may maximize survival when invaders cross environmental barriers during biological invasions. In the studied mussels, demethylation was generally higher in populations subjected to greater stress levels. This would support the idea of greater plasticity favoured through epigenetic changes in invasive species undergoing adaptations to new environments. If proven true with further case studies and experiments, this discovery would have enormous implications for the management of invasive species and native pests. For example, eradication procedures involving physical or chemical treatment and causing sub-lethal stress to target organisms can potentially induce demethylation in the survivors, allowing for higher plasticity, and likely - more aggressive invasive behaviour. Therefore, if total extirpation of the unwanted population is impossible (or impractical), smooth ecosystem-based mitigation approaches should be considered. For example, suppression of invasive population by mechanical removal^84^, targeted harvesting of species (if applicable) or biocontrol^85^, complemented with enhancement of local biotic resistance by supporting native biodiversity^84^ and improving the overall ecological status of the ecosystem^86^ could be recommended.

As a last remark, epigenetics should be explored as a tool for determining invasiveness of non-indigenous species and assessing the potential associated risks. In a world of increased interchange of species across latitudes, it is impossible to prevent introductions completely. Therefore, along with minimising the risks of new incursions, by implementing national and international legislation initiatives (like IMO Ballast Water Convention, New Zealand Craft Risk Management Standard, ICES Code of Practice on the Introductions and Transfers of Marine Organisms), an effort should be put to enable better decisions about where to invest limited resources and get the best possible outcomes for biosecurity. Perhaps, epigenetics could assist in that, providing informed advice on the species with invasive potential enhanced by specific methylation patterns, and thus of the top priority for management and rapid response measures. Suarez-Ulloa *et al*.^87^ = highlighted the importance of epigenetics as a potential tool for pollution monitoring, using marine invertebrates as model systems. This recommendation could be extended to marine biopollution by nuisance organisms. Ultimately, finding epigenetic markers specific to high-profile invaders could empower development of effective preventive frameworks and overall reduce the ecological threats caused by biopollution in the oceans worldwide.

## Electronic supplementary material

Supplementary Figure 1

Supplementary Table 1

Supplementary Table 2
