# Supplementary material for: Stress related epigenetic changes may explain opportunistic success in biological invasions in Antipode mussels
Source: Sci Rep. 2018 Jul 17;8:10793. doi: 10.1038/s41598-018-29181-4 (PMC6050280; doi:10.1038/s41598-018-29181-4)

Stress related epigenetic changes may explain opportunistic success in biological invasions in Antipode mussels

Alba Ardura[1], Laura Clusa[1], Anastasija Zaiko[2,3], Eva Garcia-Vazquez[1*], Laura Miralles[1*]

1: Department of Functional Biology, University of Oviedo. C/ Julian Claveria s/n. 33006-Oviedo, Spain

2: Coastal and Freshwater Group, Cawthron Institute, Private Bag 2, Nelson 7042, New Zealand

3: Marine Research Institute, Klaipeda University, H. Manto 84, Klaipeda 92294, Lithuania

* Co-senior authors, equal contribution in this article.

Supplementary Figure 1.

Neighbor-Joining phylogenetic tree constructed with MEGA6 software, bootstrap 10,000 and Jukes-Cantor evolutionary model (the best model obtained with MEGA6). It is based on 225 bp fro Nelson sequences (NEL) and Havelock sequeces (NZ) from this study, together with GenBank references from 4 different lineages described in Hilbish et al.[3] (AF179451.1, AF179452.1, AF179453.1 and AF179459.1).

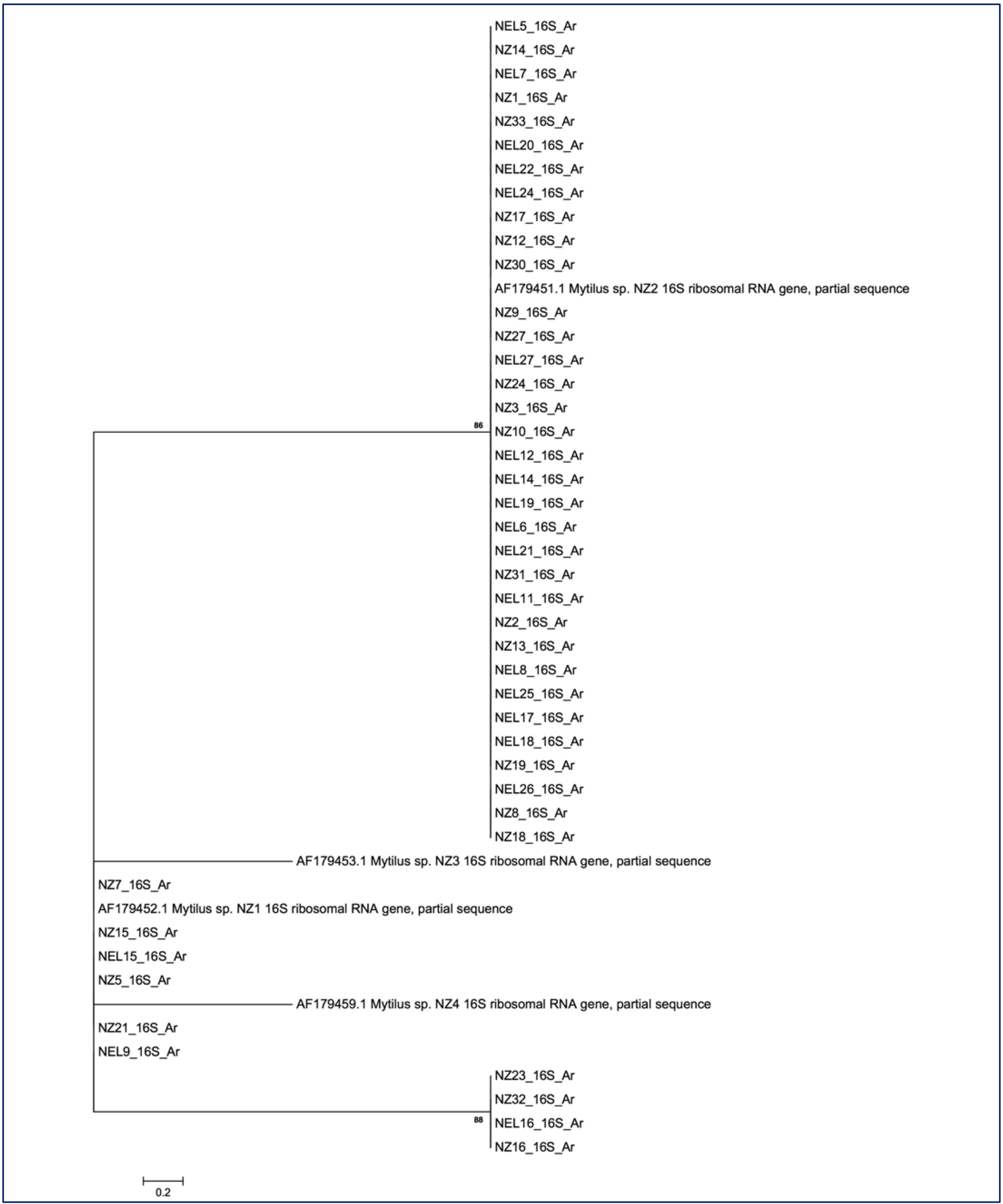

Supplement: Supplementary file 1 — Supplementary Figure 1 [file 41598_2018_29181_MOESM1_ESM.pdf]
